# The effect of herbicides on morphological features of pollen grains in *Prunus serotina* Ehrh. in the context of elimination of this invasive species from European forests

**DOI:** 10.1038/s41598-023-31010-2

**Published:** 2023-03-22

**Authors:** Dorota Wrońska–Pilarek, Irmina Maciejewska–Rutkowska, Kacper Lechowicz, Jan Bocianowski, Maria Hauke–Kowalska, Marlena Baranowska, Robert Korzeniewicz

**Affiliations:** 1 Department of Botany and Forest Habitats, Poznań University of Life Sciences, Wojska Polskiego 71d, 60-625 Poznań, Poland; 2 Department of Mathematical and Statistical Methods, Poznań University of Life Sciences, Wojska Polskiego 28, 60-637 Poznań, Poland; 3 Department of Silviculture, Poznań University of Life Sciences, Wojska Polskiego 71a, 60-625 Poznań, Poland

**Keywords:** Plant breeding, Plant reproduction, Pollen, Plant sciences, Plant stress responses, Wounding

## Abstract

*Prunus serotina* Ehrh. is an alien invasive neophyte widespread in European forests. So far, no effective methods of its elimination have been developed. For this reason, the aim of our study was to determine how herbicides affect the morphological characteristics of pollen grains. This knowledge may be crucial to control this invasive species. The current study was carried out in a research area of 2.7 ha located in the Zielonka Forest near Poznań, Poland (N 52°31′58.016″, E 17°05′55.588″). We tested morphological differences among ten features of *P. serotina* pollen, based on the samples collected from 15 control trees compared to the 50 trees treated with five different herbicides. In total 1950 pollen grains were measured. We confirmed the adopted hypotheses of long-term herbicide influence on *P. serotina* pollen. Pollen grains from the control trees had a longer equatorial axis, were more elongated in shape and had the largest range of exine thickness compared to the pollen from the herbicide-treated samples. Exine thickness in the control sample was on average 0.74 µm, ranging from 0.42 to 1.19 µm. The average values and the ranges of this trait in the samples treated with herbicides were larger (e.g. average exine thickness was from 0.90 to 0.95 µm). There were differences in the P/E ranges of variability between the control and herbicide-treated samples. In the control sample the P/E ratio was 1.32–2.04 and elongated forms of pollen shapes prevailed, while in the herbicide-treated samples it ranged from 1.03 to 1.47. The share of deformed pollen grains in the herbicide-treated samples was lower than expected, ranging from 8.7 to 25.3%, while in the control samples it was 6%. Logo and Mustang turned out to be the most effective among the herbicides used in the described research. The two used application methods were found to have an effect on pollen quality.

## Introduction

Overall, the spread of invasive plant species is intensifying as a result of progressing climate change and increasing anthropopressure. It poses a threat to the functioning of natural ecosystems, including depletion of the species composition in plant communities^1^, which consequently leads to reduction of biodiversity^2^.

The American black cherry (*Prunus serotina* Ehrh.) is an invasive plant species commonly found in European forests^3,4^. The species introduction on the European continent started in the early seventeenth century. It was initially planted in parks and gardens. At the turn of the nineteenth and twentieth centuries it began to be introduced as an admixture assumed to enrich the understory of poor, coniferous forest habitats. In addition, it was recommended as a phytomeliorative^5^ and soil-protecting species^6^. Now, in Europe *P. serotina* is classified among the hundred most invasive organism species^7^.

Currently in Poland *P. serotina* is an alien invasive kenophyte widely distributed throughout the country, most frequently in its central and south-western parts^8^. So far numerous studies on the expansion of the American black cherry into forest phytocoenoses have been carried out in Poland^9–13^. The invasive character of *P. serotina* is the result of its prior introduction into forests on a mass scale, while the constant increase in its range results, among other things, from the lack of natural “enemies”, intensive generative and vegetative propagation, and zoochory^11^.

In Europe the fight against *P. serotina* has a long history; unfortunately, no effective methods have been developed so far. Many publications recommend the use of glyphosate, which is effective; however, restrictions apply to its use and thus it requires individual approvals and permits. The use of herbicides in the control of *P. serotina* is associated with limitations due to the potential risk of environmental contamination and violations of the pesticide policy. European forestry limits the use of herbicides to control invasive species in protected areas and promotes the use of mechanical methods, despite their low effectiveness^14^. Herbicide applications near rivers and water bodies are prohibited. In addition, there is the problem of the lack of acceptance of alternative herbicide application techniques (e.g., by injection, etc.), which efficacy has been positively evaluated in experimental studies. Nevertheless, they have not been recommended by the relevant government agencies^15^. In Poland, where the state forests are managed by a single administrative body, a list of herbicides approved for use in forestry is published periodically and specifies the conditions for their use^16^. In practice, herbicides in forests are used on a small scale and the admissible formulations contain glyphosate. Restrictions on the use of plant protection products in forests result in a lack of research and registration of new environmentally safe herbicides. The registration, trade and professional use of herbicides is regulated by law and is most often subject to the supervision of national departments of agriculture. The common pesticide policy conducted in the European Union (EU) countries stands out against this background. There are uniform rules for the registration and trade of plant protection products in the EU countries (Regulation (EC) No. 1107/2009)^17^. The regulation specifies the rules for the authorization of active substances as well as the criteria and conditions that must be fulfilled by the plant protection products used. In each case, information on the application purpose, chemical composition, method of administration, and toxicity is included in the label of the approved pesticide. For many years chemicals have been used to combat American black cherry. 2,4,5-trichlorophenoxyacetic acid (2,4,5-T) was used in the 1960’s. However, its continued use was later prohibited because of its toxicity to fauna and humans^18^. Since the mid-1970s preparations containing glyphosate have been widely applied to eliminate *P. serotina* from European forests. The effect of glyphosate consists in the inhibition of the activity of enzyme 5-enolpyruvylshikimate-3-phosphate synthase, which is a key determinant for the shikimate pathway^19,20^ responsible for the synthesis of aromatic amino acids, i.e. phenylalanine, tyrosine and tryptophan^21^. The use of glyphosate at high concentrations and by different application methods results in very high efficacy (more than 90% mortality) of glyphosate in the control of American black cherry^22–26^.

The black cherry is characterised by a high potential for generative reproduction, usually it starts fruiting early, as early as the age of four years^27^. The most intensive flowering is observed before the age of 30, but the production of healthy and germinable seeds may last up to 100 years^28^. Under good light conditions flowering is abundant and can reach a maximum of 59 flowers per inflorescence, which is more than 133,000 flowers per tree^29^. Black cherry is insect pollinated and does not produce viable seeds from self-pollination^30^. Its flowers secrete a mixture of volatile organic compounds dominated by β-ocimene and several phenylpropanoids/benzenoids. The insect assemblage associated with black cherry flowers include three major orders (Coleoptera, Diptera and Hymenoptera)^31^. According to McLaughlin et al.^32^, andrenid bees (Andrenidae: Hymenoptera) are probably the most important pollinators. Fruits of *P. serotina* are consumed by several dozen species of birds and mammals^33,34^, which is one of the most important features facilitating spread of this species^35,36^. For this reason there is a need for research on the possibility of limiting generative reproduction in the fight against this invasive species. A study by Golt & Wood^37^ showed that glyphosate was present in *Rosa acicularis* tissues up to two years after its application. Moreover, it affected petal and pollen morphology in flowers in the next growing season after its use. Wrońska-Pilarek et al.^38^ proved that herbicides applied in spring affected the morphological features of inflorescences and flowers of black cherry in the next growing year. Efficacy of six tested herbicides containing various active substances in the control of *P. serotina* was demonstrated. A year after their application a decrease was observed in the size of inflorescences and the number of flowers. The current work was a continuation of the above-cited study.

The aim of this study was to determine the effect of herbicides on pollen morphology. We hypothesised that plants treated with herbicides would be characterised by morphological changes in pollen grains, which could affect generative reproduction of *P. serotina*. We expected the proportion of deformed pollen grains in samples from control trees to be smaller compared to those from treated plants. Additionally, we assumed that the method of herbicide application might influence pollen morphology. A new aspect of our research was to investigate the response of *P. serotina* pollen one year after the plant herbicide treatment depending on two application methods used, as well as describe the morphological structure of pollen grains in this species.

## Study area

The research was conducted in the Zielonka Forest near Poznań (at a distance of 33 km), in the Forest Experimental Station (division 49 c) of Murowana Goślina, which is a unit of the Poznań University of Life Sciences, Poland. The geographical coordinates of the experimental site are N 52°31′58.016″, E 17°05′55.588″ (see Supplementary Fig. S1). In 2020, employees of the Department of Silviculture, the Poznań University of Life Sciences, the 2.7-hectare black cherry research area was established under the canopy of a pine stand, where pine modelling and productivity work had been carried out^39^. Black cherry trees, of 30-years old, grew under the canopy of 97-years old Scots pine in a mixed fresh forest habitat. The height of the black cherry trees was 10–12 m and the diameter at breast height (DBH) was 8–12 m.

## Material and methods

### Herbicides used and dosing methods

The herbicides were applied on April 7, 2020 under optimal weather conditions: air temperature 10–15 °C, no rain, light wind. Two methods of herbicide application were used in the experiment. The first method was to apply herbicide solutions directly to the holes in the trunk (see Supplementary Fig. S2). Holes in the trunk were made through the bark with a drill (abbreviation d), diameter of 8 mm, at a height of 1.3 m and at an angle of approx. 45° to the trunk axis. The holes were secured against drying out with a wooden pin. The second method of application was to apply the herbicide to the trunk, in which the bark (abbreviation b) and wood were injured with a hand saw (see Supplementary Fig. S3). Spraying was carried out using a self-made pressure hand sprayer with a brush cover. Ten millilitres of herbicide solution were applied to each tree. Assuming that about 50% of the herbicide solution went directly to the wound and to the inside of the wound (by sprinkling the exposed sapwood), it resulted in a comparable amount of pure herbicide applied in both methods. The concentrations of the herbicide solutions are given in Table 1.

**Table 1:** Characteristics of herbicides used in the study.

Trade name	Abbreviation	Active substance content	Herbicide concentration First method—Holes in trunk	Herbicide concentration Second method—Wound in the trunk	Manufacturer
Chikara 25 WG	Chid – method I Chib – method II	Flazasulfuron - 25%	1.96%	0.79%	Belchim Crop. Protection, Belgium
Chwastox turbo 340 sl	Chwd – method I Chwb – method II	4-chloro-2–methylphenoxy acetic acid – 25.9%; Dicamba – 3.4%	25%	10%	CIECH Sarzyna, Poland
Logo 310 WG + Mero 842 EC	Ld – method I Lb – method II	Foramsulfuron -30.0% Iodosulfuron-methyl sodium - 1.0%	1.47% Logo 310 WG 0.1% Mero 842 EC	0.59% Logo 310 WG Mero 842 EC	Bayer SAS, France
Mustang Forte 195 SE	Md – method I Mb – method II	Florasulam – 10% Aminopyralid – 0.94% 2,4-D - 17%	10%	4%	Corteva Agriscience™ U.S
Roundup Flex 480 FL	Rd – method I Rb – method II	Glyphosate – 35.75%	50%	20%	Monsanto Europe S.A./N.V, Belgium

All the methods were carried out in accordance with European Union regulations (Regulation (EC) No. 1107/2009)^17^ and the approval of the Ministry of Agriculture and Rural Development for the use of selected herbicides in experimental studies was obtained.

### Palynological analysis

The collection of plant material for research was carried only once, on July 8th, 2021. Several, randomly selected inflorescences were gathered from the upper, sun-exposed parts of the crowns of 65 pre-selected black cherry trees growing within the study area. The collected plant material was stored in the herbarium of the Department of Botany and Forest Habitats of the Poznań University of Life Sciences (PZNF herbarium no.: 2021/1–65, determined by D. Wrońska-Pilarek). No permit to conduct the research was required.

Erdtman’s method of acetolysis (1960)^40^, standard in palynology, is invasive. It may cause pollen grains to be damaged and deformed, which affects quantitative and qualitative pollen characteristics. In the current work for five randomly selected pollen samples the results of the measurements after pollen acetolysis were compared with those of the same pollen sample not subjected to any chemical preparation. No differences were found and consequently acetolysis was discontinued. So only pollen samples not subjected to any preparation were used in biometry, as well as LM and SEM examination.

For LM observations and biometry, pollen grains from inflorescences were directly transferred into glycerin droplets on basic slides. The LM study was performed using the Levenhuk D870T microscope equipped with a camera and software to accurately measure pollen grains at a 400 × magnification. To study exine pattern morphology in SEM the pollen samples were mounted onto aluminium stubs with a double-sided adhesive tape. Stubs were sputter-coated with gold-palladium and pollen was examined and imaged using a Zeiss Evo 40.

In total, 65 samples of pollen grains were analysed. Every five of them represented 13 variants of the current experiment, including 10 with the use of five different herbicides (Chi—Chikara, Chw—Chwastox, L—Logo, M—Mustang, R—Roundup) and two methods of their application (d—drilling or b—incision) as well as three control ones. The method of application was indicated by the last letter after the abbreviation of the herbicide name. The control groups referred to the samples gathered from the trees without any treatment (K), with drilling (Kd) or bark incision (Kb), but with only pure water used instead of chemicals.

In each sample 30 pollen grains were analysed. In total, 1950 pollen grains were measured. Only mature, correctly formed pollen grains were studied. Seven quantitative features were analysed, i.e. length of polar axis (P), equatorial diameter (E), length of ectocolpus (Le), thickness of exine along the polar axis (Exp), and three ratios: P/E, Le/P and Exp/P, respectively. The pollen shape classes (P/E ratio) were adopted according to the classification proposed by Erdtman^41^: suboblate (0.75–0.88), oblate-spheroidal (0.89–0.99), spheroidal (1.00), prolate-spheroidal (1.01–1.14), subprolate (1.15–1.33), prolate (1.34–2.00) and perprolate (> 2.01). In addition, the following qualitative characters were also determined: outline, shape and exine ornamentation. Exine ornamentation type was identified based on the classification proposed by Ueda^42^. Description details of the striate exine ornamentation were based on the height and width of grooves, width of striae and the number and diameter of perforations.

In all the samples (for 100 pollen grains each) the presence of aberrant pollen grains were also analysed, and their percentage share was determined. Three categories of such pollen grains were distinguished: ruptured pollen grains, usually broken within the aperture area (A), grains with cracking within the aperture area, but not ruptured (B), malformed grains, of abnormal shapes, often as a result of exine collapse or with an unusual number of apertures (C).

Descriptive palynological terminology followed Punt et al.^43^ and Halbritter et al.^44^.

### Statistical analysis

The normality of the distributions for the studied traits was tested using Shapiro–Wilk’s normality test^45^. A multivariate analysis of variance (MANOVA) was carried out to determine the effects of herbicides and application methods, as well as effect of the herbicide × application method interaction for all the observed traits jointly. Subsequently, two-way analyses of variance (ANOVA) were carried out to determine the effects of herbicides, application methods and the herbicide × application method interaction on the variability of examined traits for each trait independently. The minimal and maximal values, arithmetic means and standard deviations of traits were calculated. Moreover, Fisher’s least significant differences (LSDs) were estimated at a significance level of *α* = 0.001. Homogeneous groups for the analysed traits were determined based on LSDs. The relationships between the observed traits were estimated by Pearson’s correlation coefficients. The results were also analysed using multivariate methods. A canonical variate analysis was applied in order to present a multi-trait assessment of the similarity of the tested combinations of herbicides and application methods in a lower number of dimensions with the least possible loss of information^46^. This facilitated graphic presentation of any variation in the combinations of herbicides and application methods in terms of all the observed traits. The Mahalanobis distance was suggested as a measure of similarity for “polytrait” combinations of herbicides and application methods^47^, the significance of which was verified by means of critical value D_*α*_ called “the least significant distance”^48^. Mahalanobis distances were calculated for the combinations of herbicides and application methods. The differences between the analysed combinations of herbicides and application methods were verified by cluster analysis using the nearest neighbour method and Euclidean distances^49^. All the analyses were conducted using the GenStat 18 statistical software package.

## Results

### General morphological description of pollen

Despite the great importance of *P. serotina* as an invasive species in European forests, its pollen morphology has not been studied so far. Therefore, we decided to describe it in detail as a starting point to further analyses.

**Figure 1:**
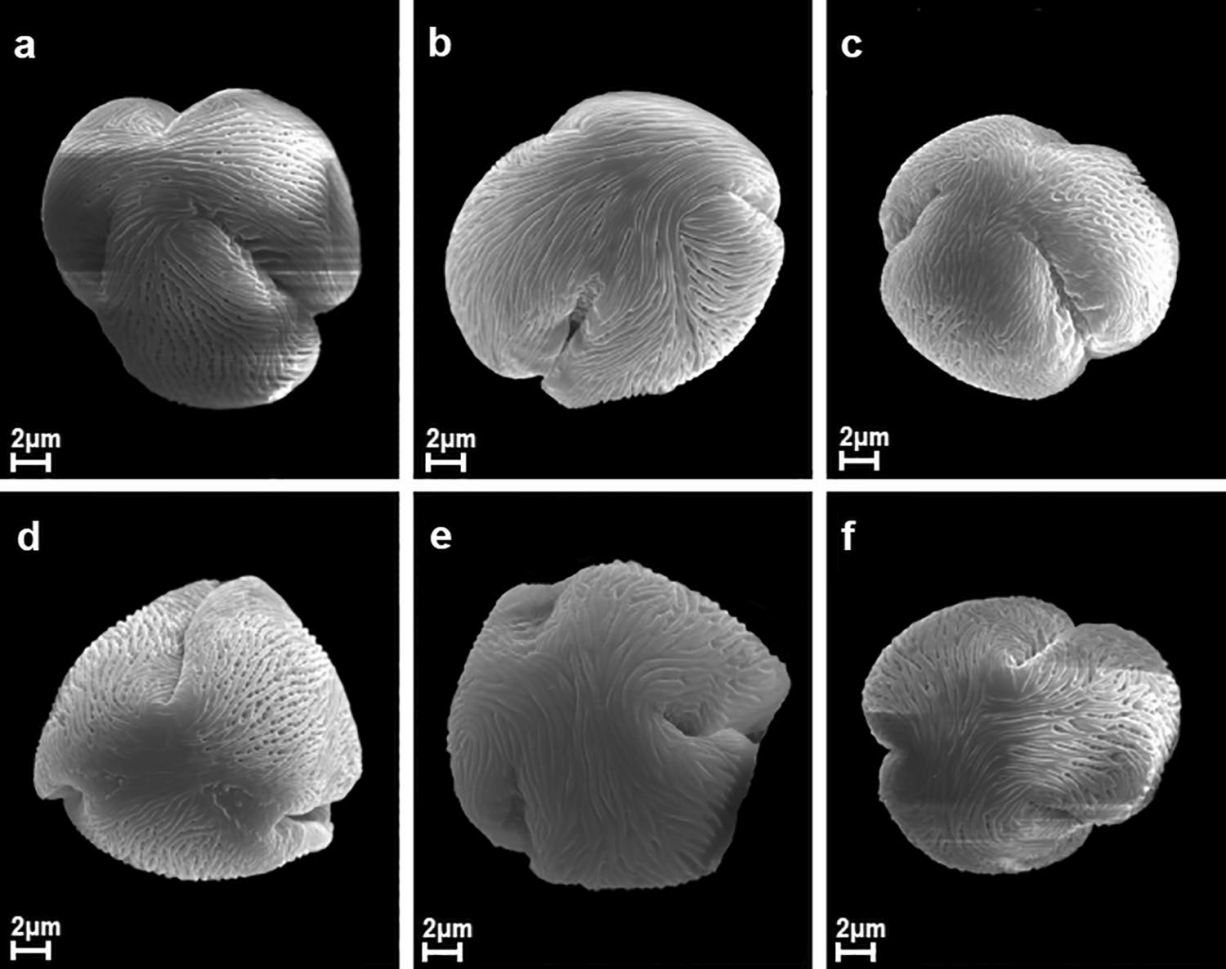
Pollen grains of *P. serotina* in polar view after application of various herbicides ((**a**) – Chikara, (**b**) – Chwastox, (**c**) – Logo, (**d**) – Mustang, (**e**) – Roundup, (**f**) – Control).

**Figure 2:**
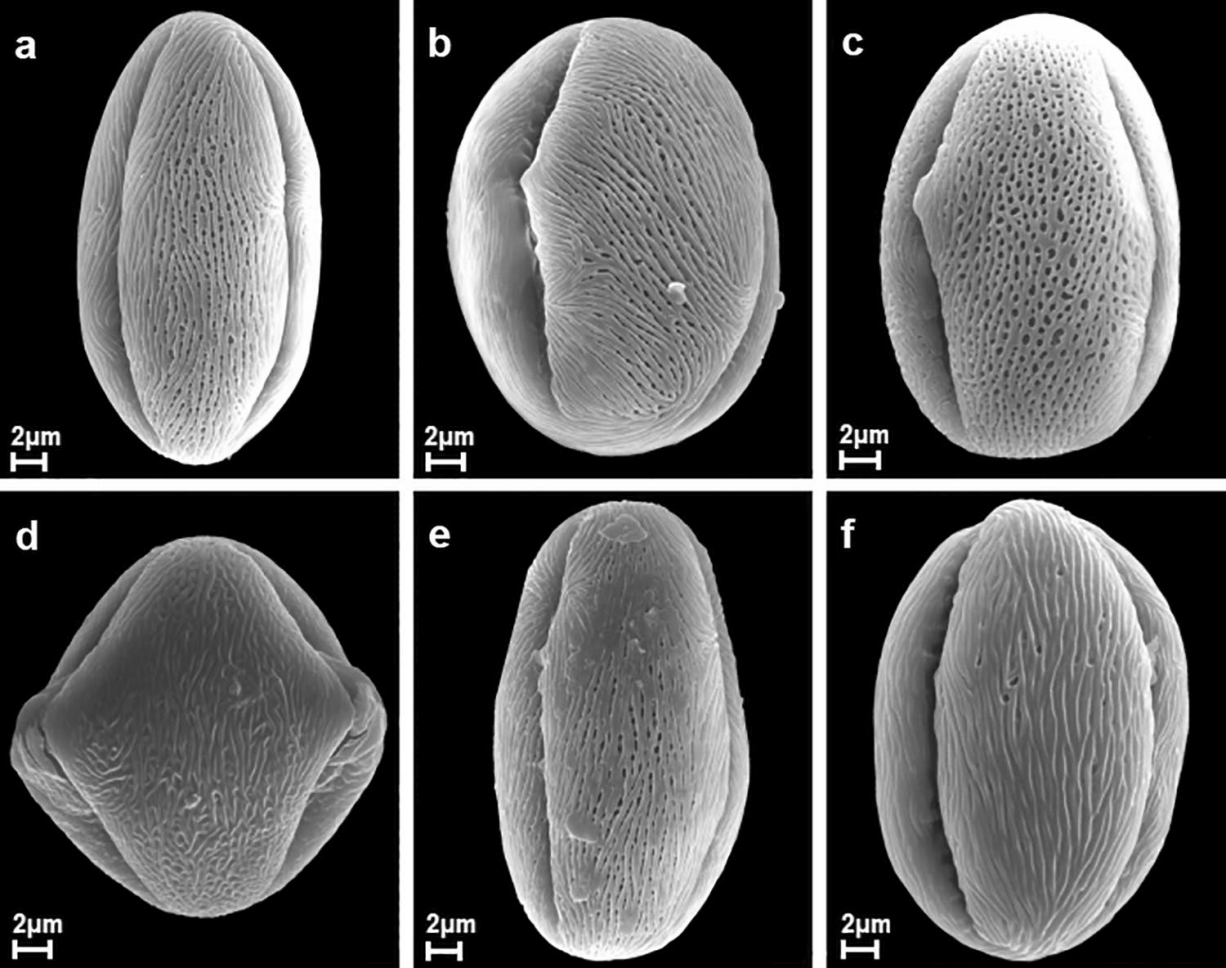
Pollen grains of *P. serotina* in equatorial view after application of various herbicides ((**a**) – Chikara, (**b**) – Chwastox, (**c**) – Logo, (**d**) – Mustang, (**e**) – Roundup, (**f**) – Control).

**Figure 3:**
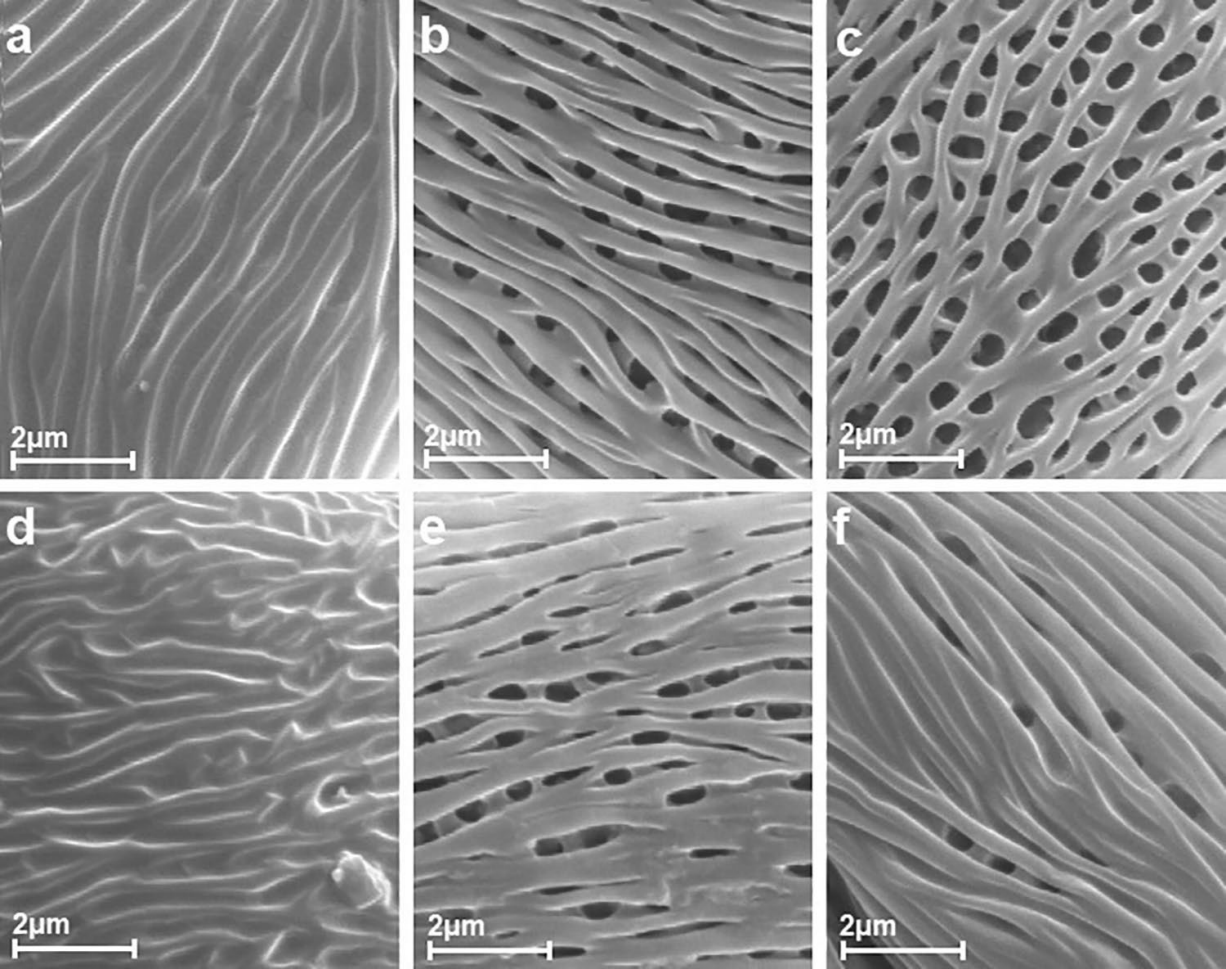
Striate exine ornamentation types of *P. serotina* after application of various herbicides ((**a**) – Chikara, (**b**) – Chwastox, (**c**) – Logo, (**d**) – Mustang, (**e**) – Roundup, (**f**) – Control).

**Figure 4:**
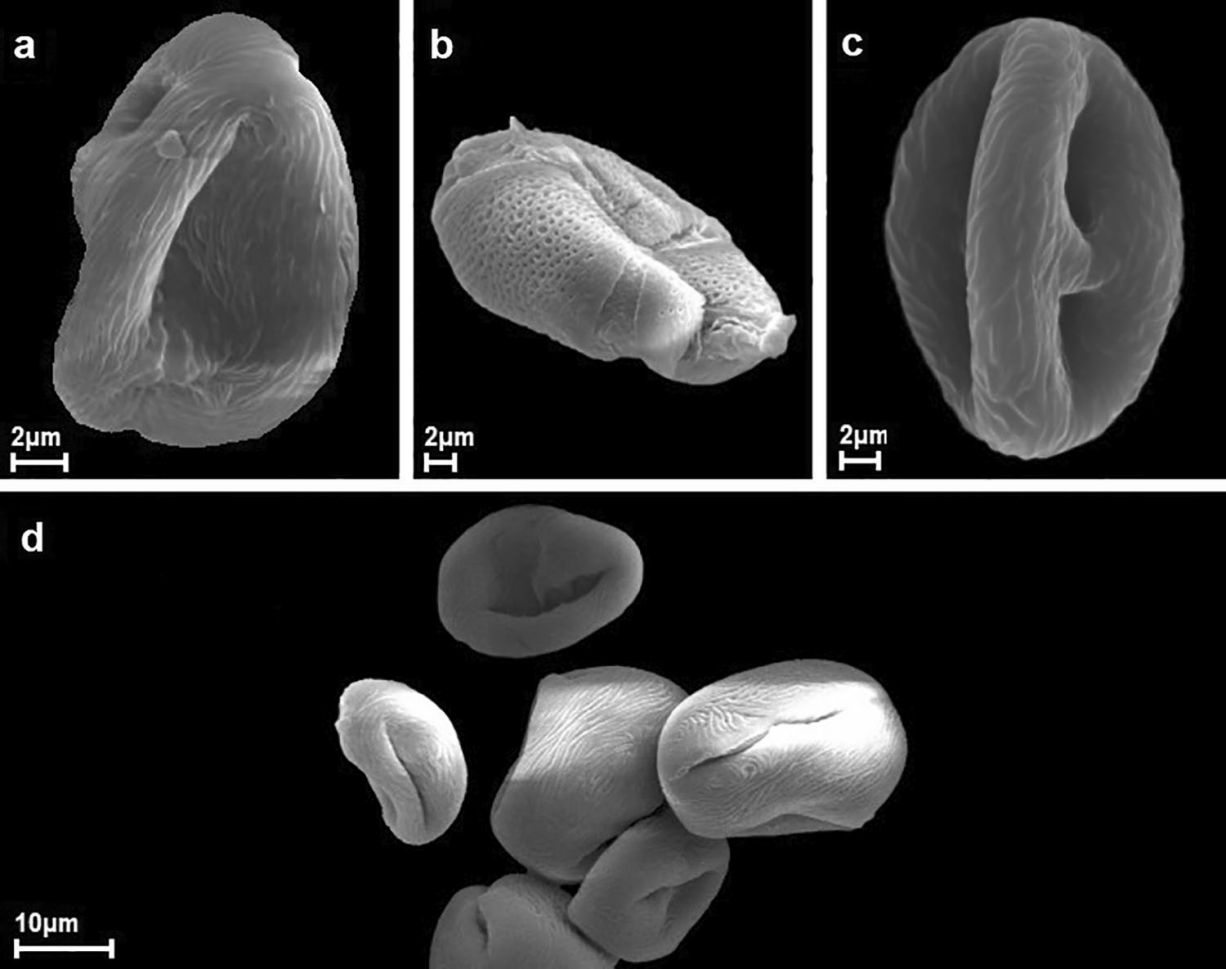
Deformations of pollen grains after application of various herbicides ((**a**) – Chwastox, (**b**) – Logo, (**c**) – Mustang, (**d**) – Roundup).

A description of pollen grains of *P. serotina* is given below and illustrated in the SEM photographs (Figs. 1, 2, 3 and 4). The morphological observations for the other quantitative characters of pollen grains are shown in Tables 2 and 3.

**Table 2:** Mean values and standard deviations for length of polar axis (P), length of ectocolpus (Le), equatorial diameter (E) and thickness of exine along the polar axis (Exp).

Trait	Length of polar axis, P			Length of ectocolpus, Le			Equatorial diameter, E			Thickness of exine along the polar axis, Exp		
Sample	Mean	Min–Max	s.d	Mean	Min–Max	s.d	Mean	Min–Max	s.d	Mean	Min–Max	s.d
K	29.48^a^	24.12–33.43	2.054	24.44^a^	19.03–29.03	2.138	17.15^f^	14.25–20.77	1.165	0.7436^e^	0.42–1.19	0.113
Kb	26.56^cde^	20.5–32.31	2.288	22.48^bcd^	18.42–28.15	2.192	23.13^bc^	18.92–27.83	2.234	0.9268^abcd^	0.72–1.08	0.093
Kd	27.02^bc^	23.32–31.63	1.839	22.92^bc^	18.53–27.79	2.05	23.81^ab^	18.5–29.23	1.971	0.9027^d^	0.61–1.1	0.114
Chib	26.62^cde^	23.41–32.39	1.833	22.89^bc^	19.44–28.64	1.909	23.37^ab^	17.53–28.93	2.718	0.9218^abcd^	0.72–1.08	0.099
Chid	25.64^fg^	21.52–31.03	1.965	21.57^ef^	17.15–26.9	1.749	23.46^ab^	18.51–27.68	1.764	0.9124 ^cd^	0.64–1.08	0.100
Chwb	25.64^fg^	20.43–29.4	2.04	21.68^def^	16.69–26.96	2.186	20.33^d^	14.87–26.75	2.697	0.7674^e^	0.45–1.16	0.181
Chwd	24.92^g^	20.05–29.29	1.592	21.37f	18.89–26.6	1.489	24.17^a^	20.15–27.98	1.47	0.9467^abc^	0.76–1.1	0.086
Lb	26.07^def^	19.15–31.68	2.685	23.15^b^	16.51–29.05	2.708	18.68^e^	14.96–25.58	1.889	0.9145^bcd^	0.53–1.22	0.126
Ld	25.43 fg	16.82–30.85	2.488	22.74^bc^	15.47–28.58	2.599	19.71^d^	15.77–24.93	1.942	0.9449^abc^	0.69–1.2	0.121
Mb	27.29^bc^	22.73–31.86	1.918	24.3^a^	15.16–29.9	2.246	18.77^e^	12.38–26.11	2.238	0.9523^abc^	0.67–1.16	0.092
Md	25.87^ef^	21.23–31.88	1.989	22.31^cde^	18.55–29.26	2.062	22.42^c^	16.29–28.09	2.845	0.9259^abcd^	0.72–1.13	0.092
Rb	26.81 ^cd^	23.37–30.97	1.742	23.26^b^	19.01–27.58	1.77	23.58^ab^	18.19–26.95	1.87	0.9563^ab^	0.71–1.1	0.093
Rd	27.78^b^	22.48–33.11	2.009	24.18^a^	19.79–31.09	2.077	23.41^ab^	14.41–28.61	2.996	0.9578^a^	0.72–1.12	0.080
LSD_0.001_	0.782			0.805			0.838			0.042		

Samples: K – trees without any treatment, Kd – trees with drilling, Kb – trees with bark incision as well as five different herbicides: Chi – Chikara, Chw – Chwastox, L – Logo, M – Mustang, R – Roundup and two methods of their application: d – drilling or b – incision.

^a–f^In columns, means followed by the same letters are not significantly different.

**Table 3:** Mean values and standard deviations for three ratios: P/E, Le/P and Exp/P, where P – length of polar axis, E – equatorial diameter, Le – length of ectocolpus, Exp – thickness of exine along the polar axis.

Trait	P/E			Le/P			Exp/P		
Sample	Mean	Min–Max	s.d	Mean	Min–Max	s.d	Mean	Min–Max	s.d
K	1.726^a^	1.3213–2.04	0.159	0.8287^f^	0.6808–0.9223	0.039	0.0253^f^	0.0139–0.0411	0.0042
Kb	1.16^def^	0.9169–1.515	0.162	0.8468^de^	0.734–0.9512	0.047	0.0352^cd^	0.0250–0.0527	0.0048
Kd	1.14^ef^	0.9408–1.441	0.102	0.8482^de^	0.6977–0.9618	0.049	0.0336^d^	0.0206–0.0461	0.0050
Chib	1.16 ^def^	0.9128–1.661	0.195	0.86^cd^	0.7691–1.0133	0.045	0.0348^cd^	0.0233–0.0440	0.0046
Chid	1.099^fg^	0.938–1.462	0.114	0.842^ef^	0.6826–0.9528	0.047	0.0358^bc^	0.0252–0.0502	0.0045
Chwb	1.286^c^	0.8901–1.866	0.213	0.8459^de^	0.6789–0.977	0.059	0.0303^e^	0.0163–0.0494	0.0083
Chwd	1.034^g^	0.8932–1.266	0.080	0.8585^cde^	0.7555–1.0308	0.048	0.0381^a^	0.0286–0.0483	0.0041
Lb	1.41^b^	1.0545–2.015	0.204	0.8872^ab^	0.8014–0.9755	0.035	0.0355^bcd^	0.0196–0.0517	0.0065
Ld	1.303^c^	1.0167–1.841	0.191	0.8933^a^	0.7902–0.9595	0.033	0.0375^ab^	0.0250–0.0492	0.0057
Mb	1.474^b^	1.0644–1.973	0.205	0.8902^a^	0.5275–1.0034	0.045	0.0350^cd^	0.0261–0.0486	0.0038
Md	1.175^de^	0.8639–1.917	0.195	0.8628^cd^	0.7328–0.9717	0.049	0.0360^bc^	0.0260–0.0446	0.0041
Rb	1.142^ef^	0.9436–1.463	0.101	0.8678^c^	0.7552–0.9663	0.042	0.0358^bc^	0.0256–0.0450	0.0041
Rd	1.21^d^	0.9395–1.884	0.210	0.8709^bc^	0.7266–0.9638	0.048	0.0347^cd^	0.0241–0.0456	0.0040
LSD_0.001_	0.065			0.017			0.002		

Samples: K – trees without any treatment, Kd – trees with drilling, Kb – trees with bark incision as well as five different herbicides: Chi – Chikara, Chw – Chwastox, L – Logo, M – Mustang, R – Roundup and two methods of their application: d – drilling or b – incision.

^a–f^In columns, means followed by the same letters are not significantly different.

Pollen grains of *P. serotina* were tricolporate, isopolar monads (Figs. 1 and 2). According to the pollen size classification by Erdtman^41^, pollen grains analysed were mostly medium (25.1–50 µm; 73.4%), rarely small (10–25 µm; 26.6%). Average values for trait P ranged from 22.94 to 31.17 μm and most of the pollen grains belonged to the upper limit of small pollen or to the lower medium-sized pollen range. Considering all the analysed pollen grains together, the mean length of the polar axis (P) was 26.55 (16.82–33.45) µm, with the most common value of 24.42 µm. The mean length of the equatorial length (E) was 21.69 (12.38–29.23) µm and the most common value for E was 18.70 µm.

The outline in the polar view was mostly lobate, with apertures infolded, whereas in the equatorial view the outline was elliptic or circular (Figs. 1 and 2). Totally, the mean P/E ratio (pollen shape) was 1.26 (0.86–2.04) and the most common value for this trait was 1.0. About 2/3 of pollen grains were subspheroidal, with prolate-spheroidal (29.8%) and subprolate (25.4%) forms being the most frequently observed subtypes of the shape class. In addition, about 1/3 of the grains were prolate in shape.

All pollen grains had three apertures – colpori. The colpori were arranged meridionally, regularly, more or less evenly spaced, with a mean length of 22.78 (15.16–29.90) µm, at the most common value of 20.75 µm. On average, the length of the colporus (Le) constituted 86 (53–97)% of the polar axis length (P). The colpori were narrow, linear or fusiform in outline. Their width was variable and usually greatest in the equatorial zone. The endoporus was not very distinct. Sculpturing of ectocolpus membranes approached rugulate, rarely almost psilate. Colpus margins were not clearly delimited, indistinctly striate to rugulate and sometimes slightly undulate. In the equatorial zone they formed pore flaps, extending across the aperture, but not always fully merging into the distinct bridge. Pore flaps were variable, of the same or unequal length.

For all the measured pollen grains the mean exine thickness was 0.75 (0.42–1.19) µm and the most often noted value for this trait was 0.79 µm. The exine thickness constituted approx. 0.03 (0.01–0.05) of the length of the polar axis. Exine ornamentation was striate-perforate. Within the central part of the mesocolpium striae and grooves ran more or less parallel to the polar axis, but sometimes curving or looping near the colporus zone or apocolpium. Striae were forked, of varying length, with smooth and obtuse ridges (Fig. 3). On average, the width of stria was about 0.34 (0.2–0.4) µm. Grooves were of variable width (range about 0.2–0.5 µm), but most often nearing the stria width. The perforations were regularly arranged in a row, in the groove bottoms were visible. They were elliptic or circular in outline, different in size. Usually, their longest diameter (parallel to the striae) ranged from 0.2 to 0.8 µm. The number of perforations calculated in 25 µm^2^ ranged between 40 and 59.

### Pollen response to herbicides used

On average pollen grains with the longest polar axes (P) were found in the control sample group (K), not subjected to any treatment. The mean P value for the control samples K1-K5 taken together was 29.48 µm. On the other hand, the damage of the bark, without the use of any herbicide (samples Kb 1–5 and Kd 1–5), reduced P length on average by approx. 2.7 µm. Compared to the control group samples, the mean length of P was below 26 µm in 23 samples treated with different herbicides. The most visible reduction of this trait was noted after treatment with Chwastox (Chwb 1–5 and Chwd 1–5) and Logo (Lb 2, 4–5 and Ld 1–2, 4–5). The weakest pollen response to P length reduction was observed in the samples after Roundup application. Within the samples treated with the same chemical, the method of its application seemed to be critical. Often the samples treated with the herbicide by drilling had lower mean P values (about 1 µm) than in the case of the other method. Only in the case of the Roundup groups, the samples subjected to debarking had on average a shorter P axis (also about 1 µm).

An interpretation of the mean values of the equatorial axis (E) is not as unambiguous as in the case of the P values. The largest values for the mean E length were observed in the Chwastox drilling (Chwd) group (24.17 µm). The smallest mean value was noted in the control sample group (K), not subjected to any treatment (17.15 µm). On the other hand, the mean values above 23 µm (and max. 24 µm) were observed in the control sample groups, but with the bark damage (Kb and Kd), as well in the Chikara (Chid and Chib) and Roundup (Rb and Rd) groups. Almost in all sample groups, with the exception of the Roundup group, the mean E lengths were greater in the case of the use of drilling.

The pollen grains with the largest mean P/E ratio (1.73) were present in the control sample group (K), not subjected to any treatment. The smallest average value of this trait (1.03) was recorded in the Chwastox drilling (Chwd) group. With the exception of the Roundup (Rd and Rb) groups, the mean P/E values were smaller when using drilling than when removing bark. This phenomenon is also found in the Kd and Kb control groups.

The control group (K) was the most homogenous in terms of shape. In total, approx. 97% of pollen grains in K1–K5 samples analysed jointly were prolate. A clear predominance of the grains elongated in shape was also observed in the Mustang, Logo and Chwastox groups, with injured bark (Mb-95.3%, Lb-90.7%, Chwb-69.4%, respectively) and in the Logo group with drilling (Ld—78.7%). In contrast, the highest proportion of pollen grains with roughly spherical shapes were found in the Chwastox group with drilling (92%) and both Chikara groups (Chid 72.0% and Chib 60.4%, respectively). For the other sample groups, including the control groups Kb and Kd, the percentage shares of more or less spherical shape types and with an elongated axis were similar.

The differences between the mean values of colpus length in the pollen sample groups observed in the study were relatively small and ranged from 21.36 µm (samples of the Chwd group treated together) to 24.44 µm (K). There was also a limited range for mean values of colpus length and equatorial axis length ratio (Le/P), as it was between 0.83 (K) and 0.89 (Lb, Ld and Mb).

On average, the pollen control group not subjected to any treatment and the Chwastox group with bark damage were characterised by the thinnest exine (K – 0.74 µm and Chwb – 0.77 µm, respectively). In the other groups of pollen grains the mean thickness of the exine ranged from 0.90 to 0.96 µm, with maximum mean values observed in both Roundup groups (Rb and Rd). The mean values of exine thickness and polar axis length ratio (Exp/P) were almost the same in all pollen sample groups and they fell within the range of 0.03–0.04.

The lowest frequency of deformed pollen grains (6%) was found in the K group samples. Compared to this group, the share of malformed grains in the other two control groups (Kb and Kd) was more than twice as high. The largest share of malformed grains (25.3%) was observed in the Chid group. It was mainly due to the presence of grains with a cracked exine. In the other group samples treated with herbicides, the share of such pollen grains was different and ranged from 8.7 (Mb) to 16.7% (Chwd). However, in the case of the same chemical, using the drilling method increased (on average about 1–4%, to even 14% in the Chikora sample groups) the proportion of abnormal pollen grains (Fig. 4).

### Intraspecific variability of pollen grains

**Figure 5:**
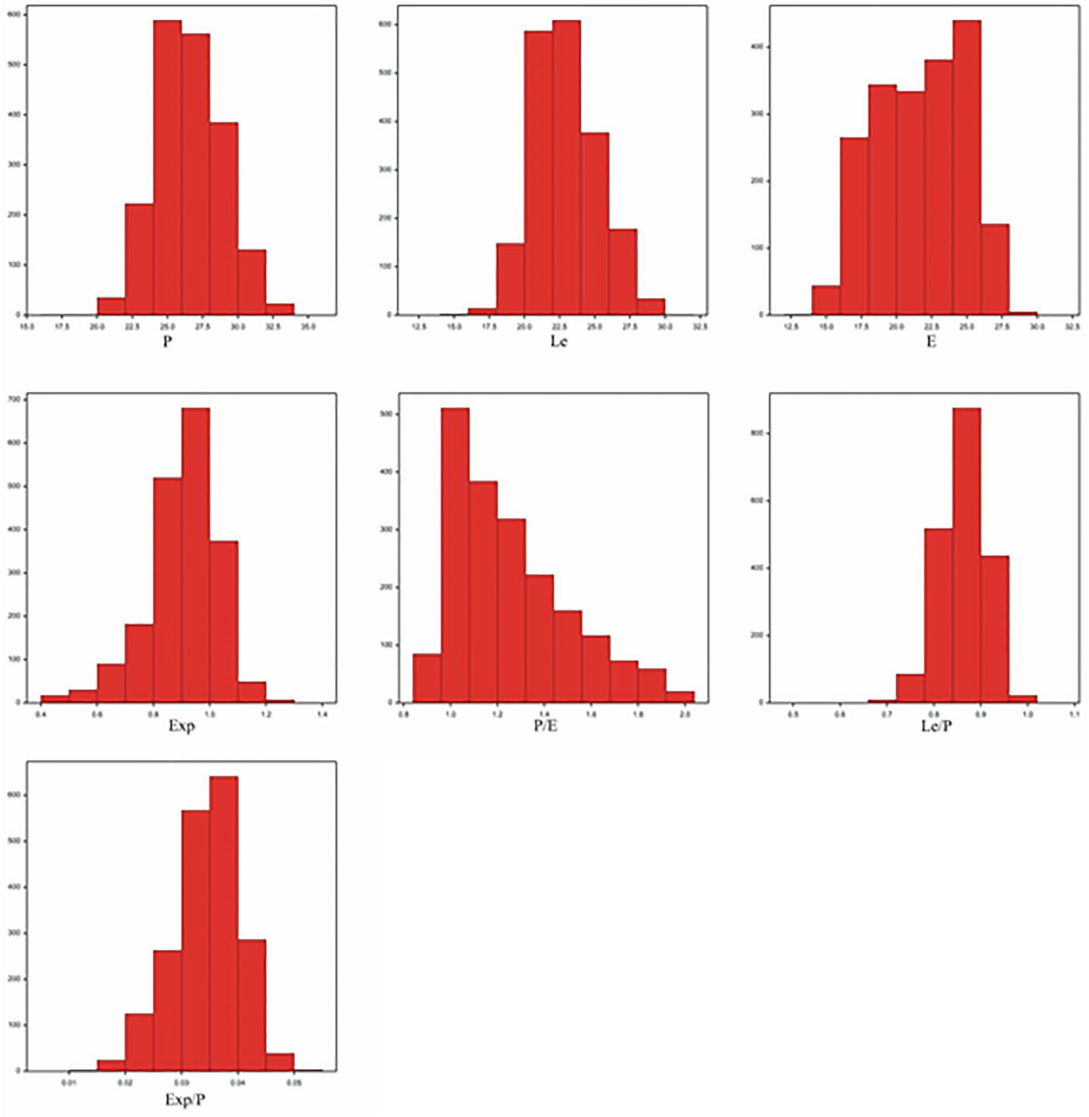
The empirical distribution of observations of the seven observed features. P – length of polar axis, E – equatorial diameter, Le – length of ectocolpus, Exp – thickness of exine along the polar axis.

The empirical distribution of observations for the seven observed traits was normal (Fig. 5). The results of the MANOVA performed indicated that the herbicides (Wilk’s λ = 0.2301; *F* = 79.35; *P* < 0.0001), application methods (Wilk’s l = 0.8890; *F* = 16.72; *P* < 0.0001) and the herbicide × application method interaction (Wilk’s l = 0.7553; *F* = 20.14; *P* < 0.0001) were significantly different when investigated in terms of all the seven traits jointly. The results of ANOVA indicated that the main effects of the herbicides and the herbicide × application method interaction were significant for all the seven observed traits. The main effects of the application methods were significant for six traits (except for Le/P).

The correlation analysis performed indicated statistically positive significant correlation coefficients between all pairs of traits (Table 4). Correlation coefficients ranged from 0.626 (between E and P/E) to 0.996 (between P and Le).

**Table 4:** Correlation coefficients between all pairs of traits.

Trait	P	Le	E	Exp	P/E	Le/P	Exp/P
P	1						
Le	0.996***	1					
E	0.887***	0.881***	1				
Exp	0.931***	0.947***	0.943***	1			
P/E	0.912***	0.913***	0.626*	0.757**	1		
Le/P	0.979***	0.987***	0.914***	0.973***	0.868***	1	
Exp/P	0.885***	0.904***	0.938***	0.989***	0.696**	0.953***	1

*P < 0.05; ** *P* < 0.01; *** *P* < 0.001; P – length of polar axis, E – equatorial diameter, Le – length of ectocolpus, Exp – thickness of exine along the polar axis.

**Figure 6:**
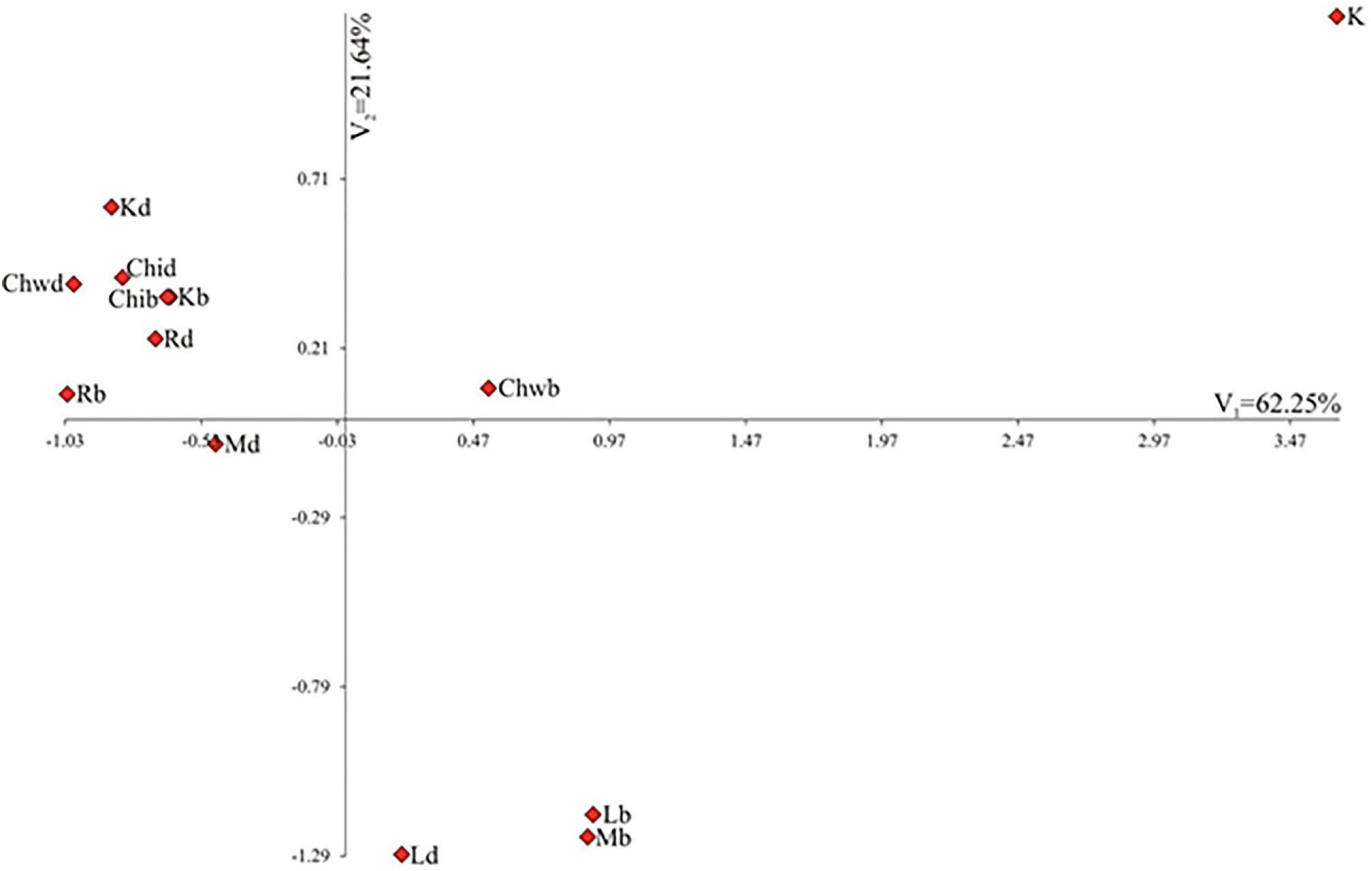
Distribution of combinations of herbicides and application methods in the space of the first canonical variable (V_1_) and the second canonical variable (V_2_). Combinations: K – trees without any treatment, Kd – trees with drilling, Kb – trees with bark incision as well as five different herbicides: Chi – Chikara, Chw – Chwastox, L – Logo, M – Mustang, R – Roundup and two methods of their application: d – drilling or b – incision.

Figure 6 shows variability of the quantitative traits for the combinations of herbicides and application methods in terms of the first two canonical variables. In the graph, the coordinates of the point for particular combinations of herbicides and application methods are the values for the first and second canonical variables, respectively. The first two canonical variables accounted for 83.89% of the total multivariate variability between the individual combinations of herbicides and application methods. Significant positive linear relationships with the first canonical variable were found for P and P/E. The first canonical variable correlated negatively with E, Exp and Exp/P. The second canonical variable was significantly negatively correlated with Le/P.

The greatest variation in terms of all the seven traits, based on the measured Mahalanobis distances, was found for combinations K and Rb (the Mahalanobis distance between them amounted to 4.81). The greatest similarity was found for combinations Kb—and Chib (0.479). The values of the Mahalanobis distances for all the pairs of combinations of herbicides and application methods are presented in Table 5.

**Table 5:** Mahalanobis distances between combinations of herbicides and application methods calculated on the basis of all seven traits.

Combination	K	Chwb	Lb	Ld	Mb	Md	Kd	Chid	Kb	Chib	Rb	Chwd
Chwb	3.714											
Lb	3.635	1.883										
Ld	4.272	1.983	0.77									
Mb	3.737	2.394	1.02	1.41								
Md	4.329	1.692	1.797	1.484	1.989							
Kd	4.568	2.048	2.577	2.295	2.679	0.996						
Chid	4.577	1.81	2.378	2.063	2.621	0.69	0.755					
Kb	4.383	1.892	2.208	1.921	2.372	0.633	0.505	0.53				
Chib	4.386	1.976	2.241	1.985	2.296	0.591	0.585	0.685	0.479			
Rb	4.81	2.326	2.39	1.994	2.391	0.854	0.695	0.981	0.685	0.601		
Chwd	4.783	2.302	2.58	2.219	2.839	0.998	1.267	0.786	1.065	0.977	1.251	
Rd	4.499	2.471	2.382	2.133	2.244	1.111	0.882	1.328	0.913	0.725	0.586	1.537

Combinations: K – trees without any treatment, Kd – trees with drilling, Kb – trees with bark incision as well as five different herbicides: Chi – Chikara, Chw – Chwastox, L – Logo, M – Mustang, R – Roundup and two methods of their application: d – drilling or b – incision.

**Figure 7:**
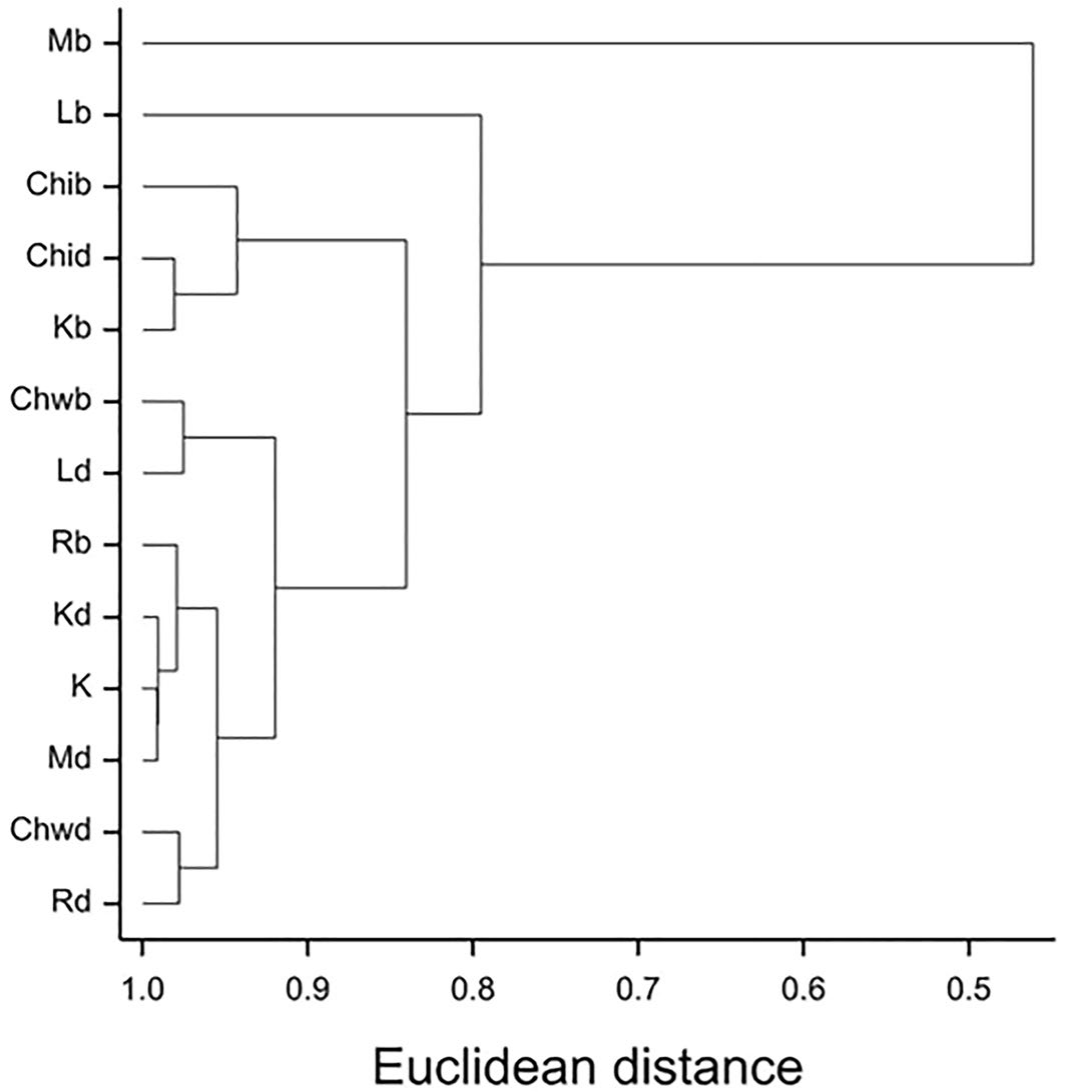
Dendrogram of cluster groupings for combinations of herbicides and application methods based on all seven quantitative traits. Combinations: K – trees without any treatment, Kd – trees with drilling, Kb – trees with bark incision as well as five different herbicides: Chi – Chikara, Chw – Chwastox, L – Logo, M – Mustang, R – Roundup and two methods of their application: d – drilling or b – incision.

In the presented dendrogram, as a result of agglomeration grouping using the Euclidean method, all the examined combinations of herbicides and application methods were divided into four groups (Fig. 7). The first group included only Mb and the second one only Lb. The third group comprised Chib, Chid and Kb, while the fourth one – all the other herbicides with both application methods (Fig. 7).

## Discussion

It is known that the organs responsible for generative reproduction are more sensitive to stress conditions than vegetative parts of plants^50–53^. For this reason, pollen is often used to assess the toxic effects of xenobiotics^54,55^. Pollen sensitivity to stress caused by pesticides, including herbicides, has already been proven many times. Both in laboratory tests and in field observations, regardless of the type of herbicide or the dose applied, viability of pollen grains decreases as a result of pesticide treatment^37,56–65^. Such experiments, however, rarely go beyond the analysis of pollen germination, ignoring the influence of the chemicals used on the quality of the pollen grains that remained alive. Surprisingly, according to Ochogavía et al.^65^ there were no significant differences in pollen morphology of *Helianthus annuus* after imazapyr treatment in the early reproductive stage compared to the control. On the other hand, it is known that other pesticides, such as fungicides, have direct negative effects on the morphological and anatomical structures of pollen. For example, pollen grains of *Lycopersicon esculentum* after nine fungicide applications (from the seedling stage to the end of flowering) showed a significant decrease in their dimensions and wall layer thickness^66^. In another experiment using fungicides, the chemicals were shown to affect pollen morphology of *Cucumis sativus*^55^. Compared to the control group, the values of some measured pollen traits increased, while pollen shape was changed. Long-term effects of pesticides, especially herbicides, on pollen quality are very rarely analysed. Our study showed the effect of five herbicides with different active substances on pollen morphology in *P. serotina* in the second year after their application. In general, we stated that residues of previously introduced chemicals had an impact on reducing the size of pollen grains, increasing exine thickness and differentiating their shape. The latest research results^67^ indicate that herbicide residues (e.g. glyphosate) can persist in different plant tissues for more than 10 years. The mechanism behind this process is not completely clear yet, but in our study it is likely an expression of the previous herbicide impact on the archesporial tissue of flowers. Pollen reactions manifested in morphological changes may not be pronounced. In general, we stated that residues of previously introduced chemicals had an impact on reducing the size of pollen grains, increasing exine thickness and differentiating their shape. However, the results of our research showed clear differences in the ranges of variability in some morphological pollen features between the control samples and samples treated with herbicides. Exine thickness in the control sample was on average 0.74 µm, ranging from 0.42 to 1.19 µm. The average values and the ranges of this trait in the samples treated with herbicides were usually larger (e.g. average exine thickness was from 0.90 to 0.95 µm). The significance of the result obtained needs to be stressed here, since proper development of the pollen tube, being critical for germination efficiency, depends on the thickness of the exine. Similarly, there were differences in the P/E ranges of variability between the control and herbicide-treated samples. In the control sample the P/E ratio was 1.32–2.04 and elongated forms of pollen shapes prevailed, while in the herbicide-treated samples it ranged from 1.03 to 1.47. The results given above are all the more valuable, because we analysed whether herbicides might affect pollen morphological traits during the second vegetation season. After such a long time, the effect of herbicides weakened and this was reflected in the differences in measurements. Still the differences we observed in exine thickness may be critical to pollen quality of *P. serotina*. For example, in an experiment on the temperature effect on the development of *Pisum sativum*^68^ it was found that heat stress caused no visible morphological differences in pollen grains or the pollen surface, but exine thickness increased. At the same time pollen germination was reduced. Similarly, during high-temperature stress experiments on soybean revealed changes in the anatomical pollen features, including a thicker exine wall, the consequence of which was a reduction in its germination capacity in vitro^69^. On the other hand, in the genus *Populus* high temperature induced 2n pollen grains with the abnormal ectexine structure, but no significant differences in germination rates of induced 2n pollen were observed^70^. Our study showed the effect of five herbicides with different active substances on pollen morphology in *P. serotina* in the second year after their application. In general, we stated that residues of previously introduced chemicals had an impact on reducing the size of pollen grains, increasing exine thickness and differentiating their shape. Our results are generally consistent with the research by Golt & Wood^37^ on *Rosa acicularis* pollen in the second year after the application of glyphosate-based herbicides. As in our work, those authors indicated significantly smaller dimensions of pollen grains in *R. acicularis* as a result of herbicide impact. Golt & Wood^37^ at herbicide-treated sites observed pollen shapes that were not present among the controls. This difference was found in about 15% of pollen grains. In our study the differences in shape between the control and the herbicide-treated samples were even more marked. The control group was the most homogenous, with almost only prolate grains, while at least 50% of shapes similar to spherical grains were recorded in the Chikara and Roundup group samples.

The main exine function is to protect reproductive cells from various environmental stresses. The exine shows remarkable resistance to microbiological, chemical and physical degradation due to sporopollenin, considered “the most resistant organic material known”^71,72^. Exine formation begins after male meiosis, when the prototypes of exine structures develop. Therefore the tapetum, as a source of precursors for the pollen wall, plays a crucial role in pollen formation and development. Changes in primexine composition caused by mutations may interfere with its ability to provide proper exine development^73^. In the ongoing research on *P. serotina*, this phenomenon undoubtedly explains the changes in its pollen morphology, such as exine thickness, occurring in the second year after herbicide use. The exine layer plays a major role in the process of adhesion between pollen and the stigma (of the pistil). Immediately after pollination, the surface of the stigma produces a pattern that binds to the exine. This reaction occurs only for pollen of the same species^74,75^. In the case of pollen grains with a modified structure, their rejection as aliens can be assumed by the stigma of flowers (of the same species). Pollen morphology directly affects pollen collections by insects. Vaissiere & Vinson^76^ noticed a reduced efficiency in cotton pollen collection mainly associated with the length of the spines on cotton pollen, which physically interfered with the pollen aggregating process used by honey bees. Deformed pollen grains with an abnormal pollen wall patterning caused poor pollen germination^69,77^. Dereboyn & Ughuz^55^ observed that under the influence of pesticides the thickness of the exine increased with a simultaneous decrease in the germination capacity of pollen. Similarly, in many previous studies during in vitro germination of pollen treated with pesticides a decrease in germination and deformation of pollen tubes were observed^78–81^.

Effective methods of combating *P. serotina* are constantly being looked for. Invasive tree species are an increasing environmental problem, as they disturb natural ecosystems around the world by displacing native species. The fight against *P. serotina* is difficult due to its high tolerance to variable soil and climatic conditions, high potential for generative reproduction, effective seed dispersal by animals, rapid growth of trees and effective regeneration after mechanical control. In the stands black cherry trees may start to bloom even at the age of about ten years, but in the open space some individuals may reach flowering maturity as early as four years of age^28^. Under optimal light conditions there are even over 133.000 flowers per tree^29^. Population expansion of the species is estimated at up to 100 m of new area per year as an effect of generative propagation^27^. In their publication Wrońska-Pilarek et al.^38^ showed that in the same experiment black cherry trees treated with herbicides had 8–9 flowers fewer than the racemes from the control trees. With so much potential for dispersal, even small changes in the pollen morphology, the number of flowers that reduce seed production seem to be significant.

*P. serotina* is a significant threat to native habitats in forests and its occurrence can be reduced with new methods being tested^23,82,83^. Chemical methods to control black cherry are highly controversial due to the use of herbicides that contain substances known to be toxic to the environment^18,58,60,84^. Additionally, the most effective ones containing glyphosate salts are suspected to be mutagenic^85^. Therefore, more attention should be paid to how chemicals are applied to limit their impact on non-target organisms. As a rule, spraying is used in practice. However, we are of the opinion that in the case of invasive tree species, other application techniques should be considered especially in forest ecosystems. According to Korzeniewicz^83^, drilling is more effective in combating *P. serotina* than partial removal of bark. In our current research we also observed that even in the second year after herbicide application, pollen grains from the trees treated by drilling herbicide application were usually smaller in size compared to those from trees with herbicides applied through damaged bark. We observed a surprising result for the samples from the control trees (Kb and Kd) with both methods of application used (b—bark and d—drilling), but with water applied instead of a herbicide. It turned out that pollen grains of the Kb and Kd groups were morphologically similar to those treated with herbicides. It seems likely that the application method itself is associated with such a high stress for the plant that the effect is similar to that after the chemical use. However, this result needs to be confirmed in long-term experiments.

## Conclusions

- For the first time we examined the response of *P. serotina* pollen to five herbicides depending on the two application methods used, while pollen morphology of this species was also described.
- We confirmed all the adopted hypotheses of long-term herbicide influence on *P. serotina* pollen. The statistical analyses revealed an interaction between the observed pollen traits, all types of herbicides used and two methods of application. In total, pollen grains from the control trees had a longer P axis, were more elongated in shape and had the largest range of exine thickness compared to the pollen from the herbicide-treated samples. Logo and Mustang turned out to be the most effective among the herbicides used in the described experiment.
- As shown by the analysis of the coefficient of variation (CV), the characteristics of pollen grains from the control trees (Kb and Kd), treated with water (instead of herbicides) applied by two methods, were more similar to those treated with herbicides than to those collected from the control trees without any interference (K). This result may have been influenced by stress caused by bark incision (method b) or trunk drilling (method d).
- As expected, a lower proportion of deformed pollen grains (6%) was recorded in the samples from the control trees (K), but the proportion of such pollen in the herbicide-applied samples was found to be lower than expected, ranging from 8.7 to 25.3%. It was also found that herbicide application method d (drilling) appeared to be more effective, as it increased the proportion of deformed pollen grains in the samples.
- Such research should be continued in the future years because of its great importance for forest management.

## Supplementary Information

Supplementary Figures.

## Data Availability

The data presented in this study are available on request from the corresponding author.
