# Supplementary material for: The effect of herbicides on morphological features of pollen grains in Prunus serotina Ehrh. in the context of elimination of this invasive species from European forests
Source: Sci Rep. 2023 Mar 22;13:4657. doi: 10.1038/s41598-023-31010-2 (PMC10033914; doi:10.1038/s41598-023-31010-2)
Supplement: Supplementary file 1 — Supplementary Figures. [file 41598_2023_31010_MOESM1_ESM.docx]

**Supplementary information**

**
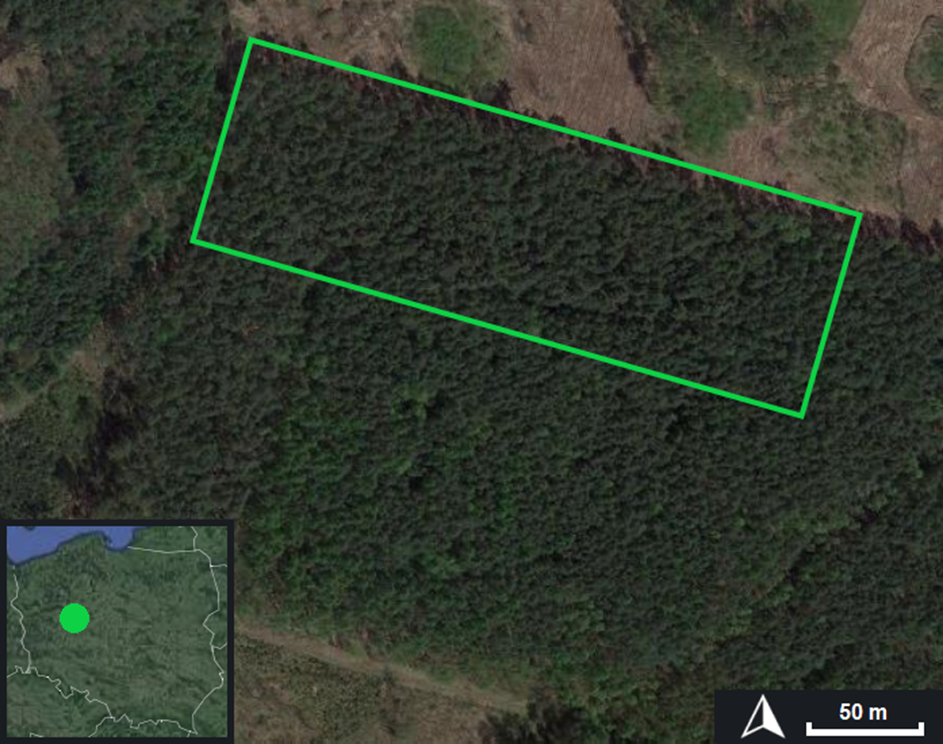
**

**Supplementary Figure S1.** Map with marked borders of the studied area. Source: Google Maps.

**
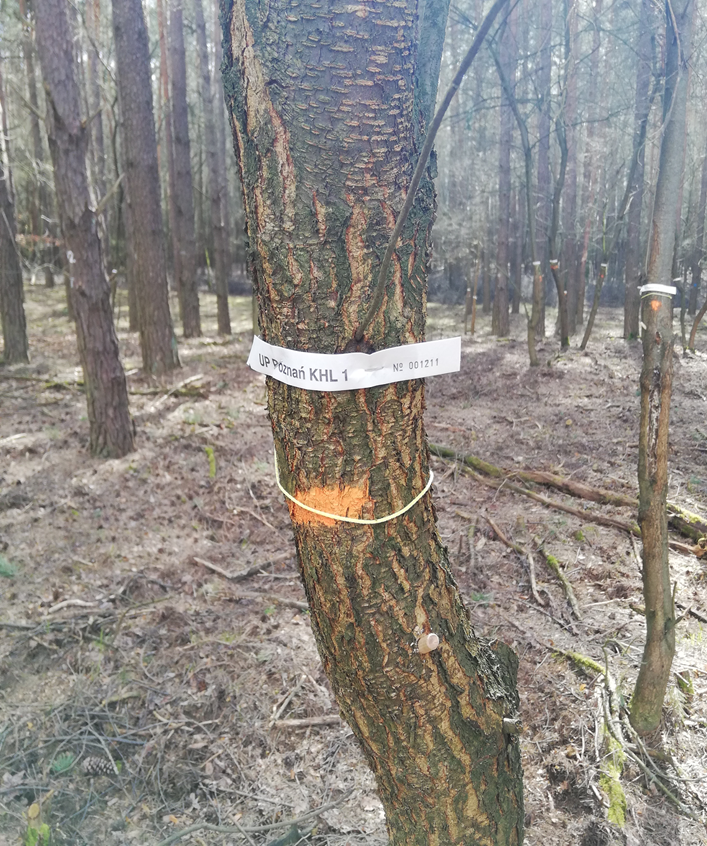
**

**Supplementary Figure S2.** The first method of application. The herbicide solutions was directly inject to the holes in the trunk.

**
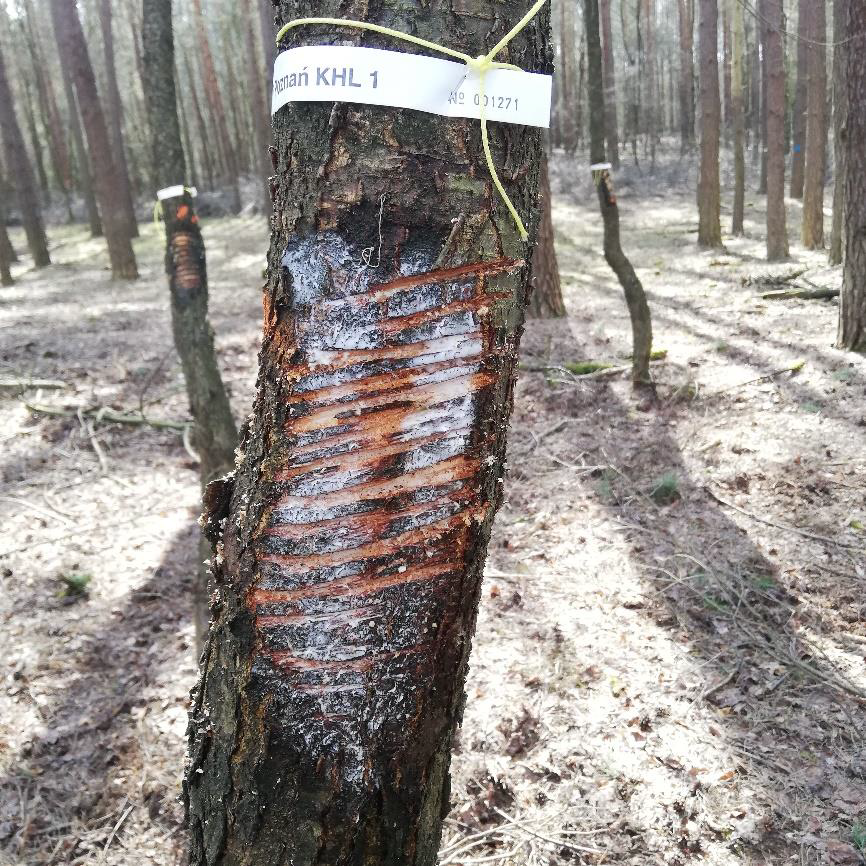
**

**Supplementary Figure S3.** The second method of application. Spraying the herbicide to the trunk in which the bark and wood were injured with a hand saw.
